# Chronic ethanol vapor exposure potentiates cardiovascular responses to acute stress in male but not in female rats

**DOI:** 10.1186/s13293-021-00371-6

**Published:** 2021-03-16

**Authors:** Paula C. Bianchi, Lucas Gomes-de-Souza, Willian Costa-Ferreira, Paola Palombo, Paulo E. Carneiro de Oliveira, Sheila A. Engi, Rodrigo M. Leão, Cleopatra S. Planeta, Carlos C. Crestani, Fabio C. Cruz

**Affiliations:** 1 Laboratory of Neuropsypharmacology, School of Pharmaceutical Sciences, São Paulo State University (UNESP), Rod. Araraquara-Jaú km 1, Araraquara, SP 14801-902 Brazil; 2 Joint Graduate Program in Physiological Sciences UFSCar/UNESP, Rod. Washington Luís km 235, São Carlos, SP 13565-905 Brazil; 3 Laboratory of Pharmacology, Paulista Medicine School, Universidade Federal de São Paulo – UNIFESP, Leal Prado Building, Botucatu 862 Street, 04024-002, Vila Clementino, São Paulo, SP Brazil; 4 Laboratory of Psychology, Psychology Department, Universidade Federal de São Carlos - UFSCar, Rod. Washington Luís km 235, São Carlos, SP 13565-905 Brazil; 5 Joint Graduate Program in Pharmacology, Pharmacology and Molecular Biology Institute - INFAR, Três de Maio 100 Street, 04044-020, Vila Clementino, São Paulo, SP Brazil; 6 Biomedical Sciences Institute, Universidade Federal de Uberlândia, Uberlândia, Minas Gerais Brazil

**Keywords:** Alcohol, Blood pressure, Heart rate, Restraint stress, Sex

## Abstract

**Background:**

Ethanol use is related to a wide variety of negative health outcomes, including cardiovascular diseases. Stress is also involved in numerous pathologies, such as cardiovascular diseases and psychiatric disorders. Sexual dimorphism is an important factor affecting cardiovascular response and has been proposed as a potential risk factor for sex-specific health problems in humans. Here, we evaluated the effect of prolonged ethanol vapor inhalation on arterial pressure, heart rate, and tail skin temperature responses to acute restraint stress, investigating differences between male and female rats.

**Methods:**

We exposed male and female Long-Evans rats to ethanol vapor for 14 h, followed by ethanol withdrawal for 10 h, for 30 consecutive days, or to room air (control groups). The animals underwent surgical implantation of a cannula into the femoral artery for assessment of arterial pressure and heart rate values. The tail skin temperature was measured as an indirect measurement of sympathetic vasomotor response.

**Results:**

Chronic ethanol vapor inhalation reduced basal heart rate in both female and male rats. Sex-related difference was observed in the decrease of tail cutaneous temperature evoked by stress, but not in the pressor and tachycardiac responses. Furthermore, prolonged ethanol inhalation enhanced the blood pressure and heart rate increase caused by acute restraint stress in male, but not in female rats. However, no effect of chronic ethanol vapor was observed in the tail cutaneous temperature response to restraint in either sex.

**Conclusion:**

Chronic ethanol vapor exposure increased the cardiovascular reactivity to stress in male, but not in female rats.

## Background

Excessive ethyl alcohol (ethanol) consumption is related to a wide variety of negative health outcomes and premature deaths [1]. According to the World Health Organization, among worldwide ethanol-related deaths in 2016, 19% were due to cardiovascular diseases [1]. Clinical and preclinical studies have demonstrated that alterations in contractile/relaxant properties of the vascular smooth muscle, changes in neuroendocrine function, impairment of baroreflex activity, and autonomic imbalance constitute important mechanisms underlying the negative cardiovascular effects of heavy ethanol consumption [1–7].

Stress is a complex and multidimensional phenomenon of great biological importance that requires an appropriate and coordinated set of physiological responses for the maintenance of homeostasis [8–11]. Restraint is one of the most commonly employed stressors to investigate stress-evoked behavioral and physiological changes in laboratory animals [12–14]. This model is characterized by unconditioned and unavoidable stress-elicited neuroendocrine and cardiovascular responses, the latter being characterized by sustained blood pressure and heart rate (HR) increases that last throughout the restraint period [10, 15, 16]. In addition, cutaneous vasoconstriction during restraint leads to a fall in the tail skin temperature [17–19].

Sexual dimorphism is an important factor affecting cardiovascular response induced by both stress and chronic ethanol access [20–27]. For example, susceptibility to hypertension in men is generally associated with increased vascular response to stress when compared to women [28]. In addition, preclinical results showed that females are more resistant than males to stress-induced cardiovascular disorders [20]. Regarding the association between ethanol and cardiovascular impairments, results have demonstrated that hypertensive effect of ethanol in men is manifested in a linear dose-dependent manner [29–31], whereas a slight protective effect of ethanol is observed in women at moderate doses [32–34]. Accordingly, studies in rodents demonstrated that high blood alcohol levels (BALs) induced by chronic ethanol consumption evoked hypertension, increased sympathetic neural activity, and enhanced baroreflex tachycardic response in males [3–5, 7], while effects considered protective to cardiovascular function were reported in females, including hypotension, increased cardiac parasympathetic dominance, and bradycardic reflex response [21, 35–38]. Regarding stress responses, decreased stress-evoked cardiovascular changes were reported following acute ethanol administration [39]. Nevertheless, the impact of chronic ethanol exposure in cardiovascular reactivity during aversive threats has never been reported.

In addition, despite the evidence of differences in cardiovascular changes related to chronic ethanol exposure between females versus males [40–46], a possible influence of sexual dimorphisms in the effect of ethanol on stress-evoked cardiovascular changes is unknown. Thus, the present study aimed to evaluate the effect of chronic ethanol vapor inhalation on blood pressure, HR, and tail skin temperature responses to acute stress, investigating differences between male and female rats.

## Methods

### Animals

We used 25 male and 27 female Long-Evans rats at post-natal day (PND 60), obtained from the animal breeding facility at the School of Pharmaceutical Sciences, São Paulo State University (UNESP) (Araraquara, SP, Brazil). These were housed in standard rat cages (plastic cages) in a temperature-controlled room at 24 °C in the Animal Facility of the Physic Institute of São Carlos, University of São Paulo (USP) (São Carlos, SP, Brazil). The animals used in this study were the same animals used in our previous work [21]. They were kept under a 12:12-h light-dark cycle (lights on between 7:00 h and 19:00 h) with food and filtered water ad libitum. Housing conditions and experimental procedures were carried out following protocols approved by the Ethical Committee for Use of Animal and Subjects of the Physic Institute of São Carlos-USP (approval# 2014/01), which complies with Brazilian and international guidelines for animal use and welfare.

### Drugs and solutions

Ethanol 95% (Labsynth, Diadema, SP, Brazil) and Isoflurane, USP (99.9% v.v). Tribromoethanol (Sigma–Aldrich, St. Louis, MO, USA) and Flunixine meglumine (Banamine®; Schering-Plough, Cotia, SP, Brazil) was dissolved in saline (NaCl 0.9%). Poly-antibiotic preparation (Pentabiotico®; Fort Dodge, Campinas, SP, Brazil) were used as provided.

### Ethanol vapor inhalation

Animals were exposed to chronic intermittent ethanol vapor in an attempt to induce a state of ethanol dependence in rodents, which is characterized by the presence of withdrawal signs, tolerance, and negative emotional symptoms upon cessation of ethanol vapor exposure [47–52]. Furthermore, compared to other methods, ethanol vapor inhalation offers advantages to the ethanol researcher, including the circumvention of rodents’ natural aversion to ethanol and the ease of maintenance of consistent BALs [47–52].

We adapted the protocol from Leão et al. [53]. Briefly, animals were housed in standard rat cages that were placed into separate sealed clear acrylic chambers (*n* = 4 per chamber), where the animals were exposed to controlled ethanol vapor. Evaporated ethanol values were adjusted as necessary to maintain animal BALs in the 150–350 (mg/dl) range. Animals were exposed daily to ethanol vapor inhalation for 14 h (7 p.m.–9 a.m.) followed by 10 h of withdrawal (no ethanol vapor inhalation), for 30 days. Blood samples were collected every week to confirm BALs. We used the single tail tip amputation (1–2 mm length per single amputation) method to perform the blood draws. Other blood collections were performed by removing the scab. The data of BALs are described in our previous study [21]. Control animals were not exposed to ethanol vapor and were not submitted to the blood drawing procedure.

### Surgical preparation

Animals were anesthetized with tribromoethanol (250 mg/kg, i.p.) and a polyethylene cannula (a 4-cm segment of PE-10 heat-bound to a 13-cm segment of PE-50) (Clay Adams, Parsippany, NJ, USA) filled with a solution of heparin (50 UI/ml, Hepamax-S®, Blausiegel, Cotia, SP, Brazil) diluted in saline (0.9% NaCl) was inserted into the abdominal aorta through femoral artery for cardiovascular recording. The catheter was tunneled under the skin and exteriorized on the animal’s dorsum. After the surgery, rats were treated with a poly-antibiotic formulation containing streptomycins and penicillins (560 mg/ml/kg, i.m.) to prevent infection and flunixin meglumine (0.5 mg/ml/kg, s.c.)—a non-steroidal anti-inflammatory drug—for postoperative analgesia.

### Blood pressure and heart rate recording

The catheter implanted into the femoral artery was connected to a pressure transducer (DPT100, Utah Medical Products Inc., Midvale, UT, USA). Pulsatile blood pressure was recorded using an amplifier (Bridge Amp, ML224, ADInstruments, Australia) and a digital acquisition board (PowerLab 4/30, ML866/P, ADInstruments, NSW, Australia). Mean arterial pressure (MAP) and HR values were derived from the pulsatile blood pressure recording.

### Tail skin temperature measurement

The cutaneous temperature of the tail was recorded using a thermal camera (IRI4010, InfraRed Integrated Systems Ltd., Northampton, UK). The temperature was measured on five points of the animal’s tail and the mean value was calculated for each recording [19, 54].

### Restraint stress

For acute restraint stress, each rat was placed in a plastic cylindrical restraint tube (diameter 6.5 cm, length 15 cm), ventilated by holes (1 cm diameter) that made up approximately 20% of the tube surface. Restraint lasted 30 min [54, 55], and immediately after the end of the stress exposure, rats were returned to their home cages. Each rat was submitted to only one session of restraint in order to avoid habituation [56, 57].

### Experimental protocols

**Fig. 1:**
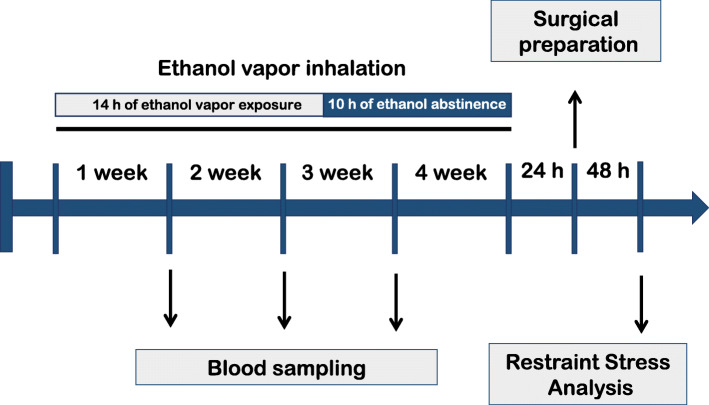
Experimental design schematic representation. Female and male rats were submitted to daily sessions of ethanol vapor inhalation for 14 h (7 p.m. to 9 a.m.) followed by a 10-h withdrawal (no ethanol vapor inhalation) to evaluate chronic ethanol vapor exposure effects on cardiovascular parameters. Blood samples (100 μl) were collected from the tip of the rat tail every week. Twenty-four hours after the last ethanol vapor exposure, we subjected all experimental groups to surgical preparation. Two days later, we performed cardiovascular measurements during basal and stress conditions (for details, see the description of “Experimental protocols” in the text)

Different sets of female and male animals were randomly allocated in four experimental groups: (i) *female control*, whose animals were kept in their home cage without ethanol vapor exposure (*n* = 8); (ii) *female ethanol vapor*, whose animals were submitted to ethanol vapor chamber daily (*n* = 9); (iii) *male control*, whose animals were kept in their home cage without ethanol vapor exposure (*n* = 5); and (iv) *male ethanol vapor*, whose animals were submitted to ethanol vapor chamber daily (*n* = 9). A schematic representation of the experimental design is presented in Fig. 1. First, animals were exposed to intermittent ethanol vapor for 4 weeks. Blood samples were collected every week to confirm BALs. Twenty-four hours after the last ethanol vapor exposure day, animals in all experimental groups were subjected to surgical preparation. The next day, rats were brought to the experimental room in their own home cages. Rats were allowed 1 h to adapt to the conditions of the experimental room, such as sound and illumination, before starting arterial pressure and HR recording. The experimental room was temperature controlled (25 °C) and was acoustically isolated from the other rooms. Cardiovascular recording of MAP and HR of freely moving rats began at least 30 min before the onset of the restraint and was performed throughout the session of stress. The tail skin temperature was measured 10, 5, and 0 min before the restraint for baseline values and at 5, 10, 15, 20, 25, and 30 min during restraint [16, 19]. At the end of the experiment, the rats were anesthetized with isoflurane inhalation and decapitated.

### Data analysis

Basal values of MAP, HR, and cutaneous temperature were evaluated by two-way ANOVA models, using sex and vapor as independent factors, and the average of the baseline measurements as a dependent variable.

The comparisons between the average values of MAP, HR, and cutaneous temperature values during the pre-stress period (basal) and restraint stress session were conducted using three-way mixed ANOVA models, with sex and vapor as between-subject factors and time (basal × stress) as a repeated-measure factor.

The time-course curves of MAP, HR, and cutaneous temperature during the stress session were evaluated by generalized estimation equation (GEE) model with sex and vapor as between-subject factors and time of stress exposure as a repeated-measure factor. GEE is a semiparametric model that evaluates longitudinal data that displays advantages when compared to repeated measures ANOVA. Contrary to ANOVA, GEE can be used when normality assumption is violated, and it achieves higher power with smaller sample sizes [58, 59]. GEE models were estimated using the robust estimator (Huber-White estimator) and a first-order autoregressive correlation matrix structure. When main effects of time, sex, or vapor were detected, data were submitted to contrast analysis with Bonferroni adjustment for multiple comparisons. When two- or three-way interactions were detected, data from different experimental groups (vapor or sex) were analyzed separately. For example, GEE detected a statistically significant interaction between sex and time for tail temperature. Therefore, as a subsequent step, data were evaluated using two GEE models: one evaluating the effect of time on males and the other on females.

ANOVA analysis was conducted on GraphPad Prism 8.0.2 software and GEE analysis was conducted on the software SPSS v. 20. For all analysis, the adopted statistical significance level (*α*) was 0.05.

## Results

### Effects of ethanol vapor exposure on basal values of arterial pressure, heart rate, and tail skin temperature

**Fig. 2:**
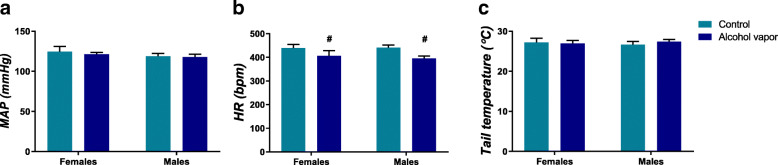
Values of **a** mean arterial pressure (MAP), **b** heart rate (HR), and **c** tail skin temperature in female and male control rats (light blue bars) or rats that were submitted to ethanol vapor exposure (dark blue bars). The bars represent mean ± SEM (*n* = 5–9 per group). Two-way ANOVA. The number sign indicates the main effect of vapor, *p* < 0.05

Figure 2 depicts the mean ± standard error of mean (SEM) of MAP, HR, and tail temperature during basal period in female and male rats. Analysis of MAP indicated no significant effect for sex (*F*_(1, 27)_ = 1.12, *p* > 0.05), vapor (*F*_(1, 27)_ = 0.21, *p* > 0.05), or interaction between factors (*F*_(1, 27)_ = 0.06, *p* > 0.05) (Fig. 2a). Analysis of HR revealed a main effect of vapor (*F*_(1, 27)_ = 5.24, *p* < 0.05), with no effect for sex (*F*_(1, 27)_ = 0.09, *p* > 0.05), or interaction between factors (*F*_(1, 27)_ = 0.13, *p* > 0.05) (Fig. 2b). No significant effects of sex (*F*_(1, 40)_ = 0.005, *p* > 0.05), vapor (*F*_(1, 40)_ = 0.10, *p* > 0.05), or interaction between factors (*F*_(1, 40)_ = 0.34, *p* > 0.05) were observed for tail skin temperature (Fig. 2c). In summary, the analysis of basal parameters showed that alcohol vapor exposure decreased HR in females and males, when compared to control groups, with no alteration of MAP and tail skin temperature.

### Effects of ethanol vapor exposure on cardiovascular responses to acute restraint stress

Independently of sex or treatment, acute restraint stress increased MAP (*F*_(1, 54)_ = 2037, *p* < 0.0001) and HR (*F*_(1, 29)_ = 1283, *p* < 0.0001) and decreased tail cutaneous temperature (*F*_(1, 43)_ = 25.14, *p* < 0.0001), as revealed by a significant effect of time (basal × stress). Thus, these alterations reflect the physiological changes promoted by restraint stress.

**Fig. 3:**
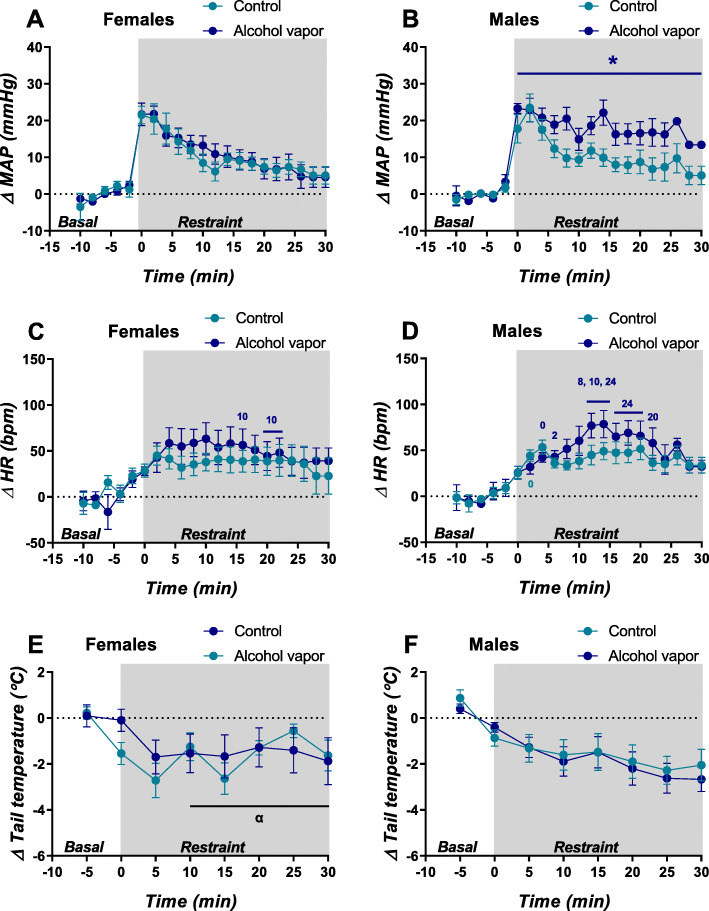
Time-course curves of mean arterial pressure (MAP), heart rate (HR), and tail skin temperature (tail temperature) during pre-stress period (basal) and restraint stress session (restraint, shaded area) in females (left) and males (right) from control (light blue lines) or ethanol vapor (dark blue lines) groups. Circles represent the mean and bars the SEM (*n* = 5–9 per group). Generalized estimation equations followed by Bonferroni multiple comparisons. (*) Different to male control group, *p* < 0.05; (0) different to time 0 of the same group, *p* < 0.05; (2) different to time 2 of the same group, *p* < 0.05; (8) different to time 8 of the same group, *p* < 0.05; (10) different to time 10 of the same group, *p* < 0.05; (24) different to time 24 of the same group, *p* < 0.05; (20) different to time 20 of the same group, *p* < 0.05. (α) Different to male groups, *p* < 0.05

During restraint stress, GEE analysis of MAP detected significant effects of sex, treatment, and time, as well as significant interactions sex × time; vapor × time; and sex × vapor × time (Table 1). Subsequent analyses revealed a significant effect of sex in the group treated with vapor (*b* = − 6.48, *p* < 0.05), but not in the control group (*b* = − 0.29, *p* > 0.05). Furthermore, a significant effect of treatment was detected in males (*b* = 7.38, *p* < 0.002), but not in females (*b* = 0.85, *p* > 0.05). Taken together, the data indicates that chronic ethanol vapor inhalation potentiates the effect of acute stress on MAP in male, but not in female rats (Fig. 3).

**Table 1:** GEE (generalized estimation equation) model with blood pressure, heart rate, or tail temperature as the dependent variable, and sex, vapor condition, and time as independent factors

Variables in the model	*W*	*gl*	*p*	
**Blood pressure**				
Sex	4.996	1	0.025	*
Vapor condition	5.795	1	0.016	*
Time	806.670	14	< 0.001	*
Sex × vapor	3.086	1	0.079	
Sex × time	155.382	14	< 0.001	*
Vapor × time	42.638	14	< 0.001	*
Sex × vapor × time	96.417	14	< 0.001	*
**Heart rate**				
Sex	0.167	1	0.683	
Vapor condition	1.304	1	0.253	
Time	447.653	14	< 0.001	*
Sex × vapor	0.003	1	0.955	
Sex × time	116.907	14	< 0.001	*
Vapor × time	88.871	14	< 0.001	*
Sex × vapor × time	62.33	14	< 0.001	*
**Tail temperature**				
Sex	0.854	1	0.355	
Vapor condition	0.033	1	0.857	
Time	42.697	6	< 0.001	*
Sex × vapor	0.195	1	0.659	
Sex × time	14.260	6	0.027	*
Vapor × time	2.746	6	0.840	
Sex × vapor × time	6.353	6	0.385	

*p < 0.05

For HR, GEE detected a main effect of time and significant interactions sex × time; vapor × time; and sex × vapor × time (Table 1). In Table 2, we presented all the significant effects of time revealed by Bonferroni pairwise comparisons. Considering the data present in Fig. 3 with the results of statistical analysis, we can conclude that males exposed to ethanol vapor exhibit considerable variation of HR during restraint, when compared to females and male control group. In this regard, male vapor group showed an increase on tachycardia response, which was maintained until the last 10 min of stress session.

**Table 2:** Summary of Bonferroni pairwise comparison of heart rate (HR) during restraint stress

Group	Bonferroni comparison	*p*	
Female vapor	Time 10 × time 16	.000	*↑
Female vapor	Time 10 × time 20	.000	*↓
Female vapor	Time 10 × time 22	.000	*↓
Male control	Time 0 × time 2	.014	*↑
Male vapor	Time 2 × time 6	.000	*↑
Male vapor	Time 8 × time 12	.000	*↑
Male vapor	Time 8 × time 14	.001	*↑
Male vapor	Time 10 × time 12	.000	*↑
Male vapor	Time 10 × time 14	.000	*↑
Male vapor	Time 12 × time 24	.000	*↓
Male vapor	Time 14 × time 24	.000	*↓
Male vapor	Time 16 × time 24	.000	*↓
Male vapor	Time 18 × time 24	.000	*↓
Male vapor	Time 18 × time 28	.026	*↓
Male vapor	Time 18 × time 30	.026	*↓
Male vapor	Time 20 × time 22	.010	*↓
Male vapor	Time 20 × time 24	.000	*↓
Male vapor	Time 26 × time 28	.004	*↓
Male vapor	Time 26 × time 30	.004	*↓

Up or down arrows indicate increase or decrease, respectively

*p < 0.05

For tail temperature, GEE detected a significant main effect of time and an interaction sex × time (Table 1). Subsequent analysis revealed a significant effect of time in both males (*W*_(6)_ = 47.45, *p* < 0.001) and females (*W*_(6)_ = 20.66, *p* < 0.05). Pairwise comparisons showed that females displayed decreased tail temperature only in the first 5 min of restraint, whereas males displayed decreased temperature throughout the stress session (Fig. 3). This result indicates an immediate decrease in cutaneous temperature, which was sustained throughout the stress period in females, whereas males showed a more gradual decline in cutaneous temperature over the entire stress session. Nevertheless, analysis did not indicate an effect of ethanol vapor in restraint-evoked skin temperature changes in either female or male animals.

## Discussion

Present findings provide evidence of the effects of chronic ethanol vapor exposure on cardiovascular function during acute restraint stress in female and male rats. Our main findings were as follows: (i) females and males exposed to ethanol vapor presented a decrease in basal HR when compared to control groups; (ii) sex-related difference was observed in the time-course of decrease in the cutaneous temperature under stress condition; (iii) chronic ethanol vapor enhanced the MAP and tachycardiac response caused by acute restraint stress in males, but not in females; and (iv) chronic ethanol vapor did not change the tail cutaneous temperature response to acute restraint stress in either sex.

Alcohol dependence is a chronic relapsing disorder characterized by frequent episodes of intoxication, preoccupation with alcohol, use of alcohol despite adverse consequences, compulsion to seek and consume alcohol, loss of control in limiting alcohol intake, and emergence of a negative emotional state in the absence of the drug [60–62]. In our study, we exposed rats to chronic intermittent alcohol vapor to model the human condition in which alcohol exposure occurs in a series of extended exposures followed by periods of withdrawal. Previous studies using the chronic intermittent alcohol vapor model showed that motivational symptoms of dependence are present in rodents at acute withdrawal time points, as evidenced by increased anxiety-like behavior, increased alcohol drinking (i.e., escalation of ethanol self-administration), and increased willingness to work for alcohol, revealing alcohol’s negative reinforcing properties [47, 63–66].

Excessive alcohol consumption is related to a wide variety of negative health outcomes, such as cardiovascular diseases [1]. An association between chronic ethanol consumption and hypertension in males is well documented [2–7, 67]. However, we did not observe alterations in MAP values, neither in males nor females. As demonstrated previously by our group [21], 4 weeks of ethanol vapor exposure was not enough to promote an increase in basal blood pressure in male rats. Previous studies have provided evidence that long-term ethanol exposure (e.g., 6, 8, or 12 weeks) is required to induce hypertension in males with BAL levels (100 to 300 mg/dl) reached during ethanol inhalation [2, 5, 67, 68]. In addition, different from other models of chronic forced ethanol exposure, such as liquid diet or ethanol in drinking water, the ethanol vapor is an intermittent model of ethanol access. In agreement with our results, Engi et al. [69] showed that 6 weeks of intermittent voluntary ethanol consumption did not induce an increase in blood pressure in male rats.

We observed decreased basal HR values in females and males exposed to ethanol vapor. The resting bradycardia effect of ethanol in females was described by other authors [21, 70–72] and might be followed by reductions in cardiac output and contractile force. For example, El-Mas and colleagues [70, 71] showed that females exposed to ethanol presented upregulation of cardiac nitric oxide synthase, which resulted in reductions of cardiac output. Further, Duan et al. [72] observed that ethanol metabolic product acetaldehyde induced cardiac contractile depression in females. Another explanation for the decrease in HR could be related to enhanced cardiac parasympathetic activity, which was observed in our previous study in females exposed to alcohol vapor [21]. In addition, it was demonstrated that decreases in basal HR are associated with inadequate tissue perfusion, arrhythmias, higher mortality, and sudden death [73, 74]. Although previous studies did not report resting bradycardia in male rodents [2, 4, 5, 7, 21], similar mechanisms and consequences to those identified in females might be related to the HR decrease identified in the present study in males.

Our findings are in line with previous studies that reported blood pressure and HR increases, and decrease in the skin temperature as physiological changes during restraint stress [10, 16, 18, 19, 75–77]. However, in contrast to other authors [22, 23], we did not observe an influence of sex on MAP and HR during the stress session. Comparing the responses of males and females under immobilization stress, Anishchenko et al. [22] observed higher amplitude and duration of MAP elevation in males and a severe tachycardia in females. Another study, using spontaneously hypertensive rats, showed a greater change in MAP in response to restraint (60 min) in males, but not in females [23]. Nevertheless, we observed a sex-related difference in the cutaneous vasoconstriction response under stress conditions. In this regard, we observed that females presented an initial decrease in cutaneous temperature, which was sustained across stress exposure, whereas males showed a more gradual decline in cutaneous temperature over the entire stress session.

This sex-related effect on sympathetic vasomotor response in cutaneous bed can be related to ovarian hormones. For instance, Zhen and colleagues [78] reported that under adrenergic nerve stimulation, arteries from females were less responsive than arteries from male rats. They also observed that this sex difference was abolished after ovariectomy of the females but not after orchidectomy of the males [78], suggesting that circulating ovarian hormones inhibits sympathetically mediated vasoconstriction. In addition, a vasodilator effect of estrogens has been reported, which is mediated by rapid stimulation of endothelial nitric oxide synthase (eNOS), via membrane-associated estrogen receptors (ERs), increasing nitric oxide (NO) production [46, 79–81]. Considering these effects, estrogen could mediate the sexual differences on hemodynamic adjustments during aversive threats.

Although ethanol exposure did not change the basal MAP in male rats, chronic ethanol vapor inhalation potentiated the effect of acute stress on MAP in males, but not in females. The arterial pressure rise during stress is mediated by an increase in vascular sympathetic tone and activation of α_1_-adrenoreceptors in vascular smooth muscle [82, 83]. In this sense, a sympathoexcitatory effect of ethanol in male rats was described in numerous studies [4, 5, 7, 67, 84, 85]. For example, Russ and colleagues [5] showed that chronic ethanol increased, via central nervous system modulation, the basal firing rate of sympathetic nerve fibers. They also observed that the sympathetic nervous activity was increased prior to the development of hypertension in males [5]. In addition, higher levels of plasma concentrations of adrenaline and noradrenaline [6, 86], as well as enhanced vascular reactivity to α_1_-adrenoceptor agonists [87–90] were observed in male rats chronically treated with ethanol. In this regard, Stewart and Kennedy [89] showed an ethanol-associated increase in the maximal contractile response to phenylephrine in endothelium-denuded preparations of male, but not of female rats. Thus, it is possible that the initial vasodilatory effect of ethanol [91, 92] is completely suppressed by increased sympathetic nervous activity in males [5, 93]. Taken together, these results could explain the increase in cardiovascular stress reactivity in male, but not in female rats observed in the present study.

The exacerbated increase in MAP in male rats exposed to chronic ethanol vapor was accompanied by an enhanced tachycardia response. Cardiac sympathetic blockers abolish tachycardia response evoked by stress, whereas cardiac parasympathetic blocker increases it [15, 77, 82, 83]. These results demonstrated that both sympathetic and parasympathetic outflows to the heart are activated during stress. Thus, an increase in restraint-evoked HR rise following ethanol exposure may result from a facilitation of cardiac sympathetic response or inhibition of parasympathetic activity in males. In this sense, the sympathoexcitatory effect of ethanol stated above might also mediate the enhanced tachycardia to stress in addition to the change on pressor response. Although previous results from our group did not indicate changes in parasympathetic activity in male rats subjected to chronic ethanol inhalation [21], we cannot exclude the possibility of an involvement of inhibition of this autonomic branch in facilitation of tachycardia to restraint.

There is significant report evidence that ethanol withdrawal is characterized by elevated glucocorticoid levels that reflect increased hypothalamic-pituitary-adrenal (HPA) axis activity, as well as by increased activity of the sympathetic nervous system, which produces significant physiological symptoms, including tachycardia, elevated blood pressure, and body temperature dysregulation [94–96]. In this regard, studies have demonstrated that dependence models involving chronic intermittent alcohol exposure constitute potent stressors, as evidenced by initial activation and subsequent dysregulation of HPA axis activity [94, 97]. Furthermore, it was demonstrated that the post-dependent state in laboratory animals is characterized by a persistently upregulated behavioral sensitivity to stress [98–100]. Clinical studies demonstrated that despite the fact that autonomic-related symptoms can return to normal in a few days during acute alcohol withdrawal in alcoholics, some cardiovascular changes may persist, especially when assessed following a stress challenge [101–103]. Our results in male rats are in accordance with these findings, since the intermittent alcohol vapor exposition intensified the cardiovascular stress reactivity.

Differently from males, no effect of chronic ethanol vapor was observed on MAP or HR values during restraint stress in females. Sexual dimorphism influences the cardiovascular effects promoted by chronic ethanol exposure [21, 89], which may impact the autonomic and hemodynamic stress responses. In contrast to sympathoexcitatory effects of ethanol in males, an increase in cardiac parasympathetic dominance has been observed in females following long-term ethanol access [21, 36, 37, 104, 105]. In fact, as stated above, we demonstrated increased cardiac parasympathetic activity in female but not in male rats exposed to chronic ethanol vapor inhalation [21]. Furthermore, other studies observed that this effect on cardiac vagal tone was estrogen-dependent [36, 104, 105]. For example, El-mas and Abdel-rahman [36] showed that the parasympathetic overactivity induced by chronic ethanol was exacerbated in estrogen-replaced ovariectomized rats, when compared to ovariectomized ones. Accordingly, El-mas and Abdel-rahman [37] observed that ovariectomized rats exposed to ethanol presented enhanced sympathetic activity as indicated by significant increases in plasma norepinephrine levels. Considering the importance of baroreflex response for the control of autonomic activity [106], we previously observed that only females exposed to alcohol vapor had increased baroreflex bradycardic response [21]. Mohamed et al. [107] demonstrated that females with intact ovaries showed an increase in bradycardic response to phenylephrine compared to ovariectomized females. It was reported that alcohol intake increases estradiol levels in humans and in rodents [108, 109], which might explain the predominance of vagal tone in females exposed to alcohol vapor and, consequently, may constitute a prominent adaptative mechanism in females that precludes the occurrence of changes in cardiovascular reactivity during stressful situations.

In addition to the evidence of female sex hormones, many recent studies indicated the positive role of male sex hormones (androgens) in cardiovascular protection [81, 110–114]. Preclinical studies showed that testosterone induces endothelium-independent relaxation in isolated coronary artery and aorta, and also contributes to the vagal outflow, but not to the sympathetic outflow to the heart of male rats [111, 113, 115]. Indeed, Ward and Abdel-Rahman [116] demonstrated that orchiectomy or androgen receptor blockade attenuates baroreflex-mediated bradycardia in rats, suggesting that androgens (including testosterone) may enhance baroreflex bradycardia via the androgen receptor at the level of the baroreceptors at the aorta and carotid, the central nervous system, or the heart. Clinical trials showed that acute administration of testosterone in patients with chronic heart failure reduced peripheral vascular resistance, cardiac afterload, and increased cardiac index [117]. On the other hand, a negative correlation has been reported between ethanol consumption and testosterone release. Several studies have shown that ethanol is a testicular toxin and it causes deficiency in testosterone secretion and spermatogenesis [118–121]. For example, Maneesh and colleagues [118] observed that serum testosterone levels in alcoholics were negatively correlated with duration of alcohol abuse. Accordingly, studies in laboratory animals showed that high doses of alcohol decreased the testosterone concentrations in alcohol-preferring and non-preferring rats [122–124]. Taking these studies into account, we may suggest that ethanol-induced reduction on testosterone levels can disrupt the cardiovascular protection of androgens, contributing to the enhanced restraint-evoked sympathoexcitatory effect in males exposed to chronic alcohol vapor.

## Perspectives and significance

Sexual dimorphism is an important factor affecting the cardiovascular response induced by both stress and chronic ethanol access. To the best of our knowledge, the findings reported here are the first to provide evidence related to the influence of sexual dimorphisms on the effect of chronic intermittent ethanol exposure on stress-evoked cardiovascular changes. Thus, the present study aimed to evaluate the effect of chronic ethanol vapor inhalation on blood pressure, HR, and tail skin temperature responses to acute stress, investigating differences between male and female rats.

Our data support the notion that exposure to chronic ethanol potentiates the cardiovascular reactivity to stressful stimuli in males but not in females and this opens doors for future work aiming at testing the mechanism underlying sex differences in cardiovascular responses to stress after a chronic exposure to ethanol. This evidence could be of great importance for implementing different strategies for treating cardiovascular diseases in abstinent alcoholic patients, considering the impact of biological sex and hormonal status in response to therapy.

## Conclusions

The results reported here showed that chronic ethanol vapor inhalation enhanced both blood pressure and tachycardiac responses to acute restraint stress in male, but not female rats. Furthermore, a sex-related difference was observed in the cutaneous vasoconstriction response during stress, as males showed a decrease in cutaneous temperature that was sustained throughout the stress session, while females presented this reduction only at the beginning of restraint. Finally, more research is necessary to improve the understanding of the impact of prolonged ethanol exposure, and the influence of sexual dimorphisms, in other physiological and behavioral responses to stress.

## Data Availability

All data are available from the corresponding author upon request.
